# Foxp3-modified bone marrow mesenchymal stem cells promotes liver allograft tolerance through the generation of regulatory T cells in rats

**DOI:** 10.1186/s12967-015-0638-2

**Published:** 2015-08-21

**Authors:** Haizhi Qi, Guangshun Chen, Yaxun Huang, Zhongzhou Si, Jiequn Li

**Affiliations:** Department of Organ Transplantation, Second Xiangya Hospital, Central South University, 139 RenMin Road, Changsha, Hunan China

**Keywords:** Gene transfer, Transcription factor forkhead box p3, Mesenchymal stem cells, Tolerance, Regulatory T cells

## Abstract

**Background:**

The transcription factor forkhead box P3 (Foxp3) is a master regulatory gene necessary for the development and function of CD4^+^CD25^+^ regulatory T cells (Tregs). Mesenchymal stem cells (MSC) have recently emerged as promising candidates for cell-based immunosuppression/tolerance induction protocols. Thus, we hypothesized that MSC-based Foxp3 gene therapy would improve immunosuppressive capacity of MSC and induce donor-specific allograft tolerance in rat’s liver allograft model.

**Methods:**

The present study utilized a lentivirus vector to overexpress the therapeutic gene Foxp3 on MSC. In vivo, Injections of 2 × 10^6^ MSC, FUGW-MSC or Foxp3-MSC into the portal vein were carried out immediately after liver transplantation.

**Results:**

Successful gene transfer of Foxp3 in MSC was achieved by lentivirus carrying Foxp3 and Foxp3-MSC engraftment in liver allograft was confirmed by fluorescence microscopy. Foxp3-MSC treatment significantly inhibited the proliferation of allogeneic ACI CD4^+^ T cells to splenocytes (SC) from the same donor strain or third-party BN rat compared with MSC. Foxp3-MSC suppressive effect on the proliferation of CD4^+^ T cells is contact dependent and associated with Programmed death ligand 1(PD-L1) upregulation in MSC. Co-culture of CD4^+^ T cells with Foxp3-MSC results in a shift towards a Tregs phenotype. More importantly, Foxp3-MSC monotherapy achieved donor-specific liver allograft tolerance and generated a state of CD4^+^CD25^+^Foxp3^+^ Tregs-dependent tolerance.

**Conclusion:**

Foxp3-engineered MSC therapy seems to be a promising and attractive cell therapy approach for inducing immunosuppression or transplant tolerance.

## Background

Transplant tolerance describes immunological unresponsiveness to donor alloantigens without the need for long-term administration of immunosuppressive drugs, while immune responsiveness to pathogens and malignant cells is maintained [1]. It is a major goal in transplantation. Gene therapy, in which malfunctioning cells are repaired by mending their DNA, offers an elegant solution to induce transplant tolerance. However, many potential problems associated with this modality of treatment have been encountered, including difficulty in regulating transgene expression, safety concerns about malignancy, infection, and inflammation [2, 3]. Determining a safe and efficient means of gene delivery and finding an optimal gene for such are believed to be keys to solving these problems.

Mesenchymal stem cells (MSC) are considered as adult stem cells with the greatest therapeutic potential because of their characteristics, which include plasticity, migratory ability, paracrine activity, and immunosuppressive activity [4, 5]. This potential may be amplified by transforming them with genes that will improve their therapeutic ability [4, 6]. MSC have recently emerged as promising candidates for cell-based immunotherapy [7–9]. MSC can inhibit immune responses and influence all components of the immune system as shown for T-, B-, natural killer-, monocytic and dendritic cells in vitro and in vivo [5, 10]. With regard to solid organ transplantation, MSC were reported to prolong skin allograft survival in baboons [11], heart [7, 8], liver [9], renal [12] and islet [13] graft survival in rodent transplantation models. In addition, MSC were already successfully employed in several clinical trials [14, 15] most notably to ameliorate steroid-resistant graft-versus-host disease in a phase II study [16]. Importantly, the immunoregulatory effects of MSC are associated with the expansion of Foxp3^+^ regulatory T cells (Tregs) [7–9, 12] and in some cases to generate a state of Foxp3^+^ Tregs-dependent tolerance [7, 8, 12, 15]. All of these studies elegantly show the importance of Foxp3^+^ Tregs in MSC induced tolerance using Foxp3^+^ Tregs depletion or adoptive transfer of Foxp3^+^ Tregs studies [7, 8, 12, 15].

Tregs are a CD4^+^ T cell population essential for maintaining self-tolerance and immune homeostasis [17, 18]. The role for Foxp3^+^ Tregs in allograft tolerance has received strong support from observations, including enhanced graft survival by adoptive transfer of Tregs populations [19, 20], the presence of small numbers of Tregs within the graft [21, 22] and peripheral induction of Foxp3^+^ Tregs following a tolerogenic protocol [23, 24]. A recent study provided definite proof that Foxp3^+^ Tregs are required not only to induce, but also to maintain the tolerant state [25]. Therefore, therapeutic strategies to induce and expand Foxp3^+^ Tregs in vivo may provide an attractive approach to guide the immune reconstitution towards tolerance.

The transcription factor forkhead box P3 (Foxp3), which encodes a forkhead/winged helix transcription factor known as scurfin, is a known master regulatory gene for the development and function of CD4^+^CD25^+^ Tregs that plays a vital role in both natural and induced Tregs differentiation from noncommitted precursors [18, 26]. Previous studies have shown that Foxp3 is expressed specifically by Foxp3^+^ Tregs in the thymus and periphery of normal mice and humans, and its expression cannot be induced in naive T cells simply by activation [27, 28]. The retroviral transfer of Foxp3 converts naive T cells into a Tregs phenotype and function [27]. Moreover, recent studies have reported a large number of key target genes which were regulated directly by Foxp3 during T stimulation, such as CD25, programmed cell death-1(PD-1)/programmed death ligand 1(PD-L1), CD28 and neuropilin-1 (Nrp-1), these target genes are the key modulators of immunosuppression [29, 30]. Based on these data, we utilized the characteristics of Foxp3 and MSC, then overexpressed Foxp3 in MSC through lentivirus carrying human Foxp3. We hypothesized that overexpression of Foxp3 in MSC could improve immunosuppressive capacity of MSC and induce donor-specific allograft tolerance in rat’s liver allograft model.

## Methods

This study was carried out in strict accordance with the recommendations in the Guide for the Care and Use of Laboratory Animals (National Institutes of Health). The protocol was approved by the Animal Ethics Committee of Central South University. All surgeries were performed under sodium pentobarbital anesthesia, and all efforts were made to minimize animal suffering.

### Isolation, culture, and characterization of MSC

Isolation and primary culture of MSC were performed according to previously reported method [31]. Isolated MSC were routinely characterized inhouse by flow cytometry for their cell surface markers, and differentiation of MSC into adipocytes, chondrocytes and osteoblasts in vitro was performed as previously described [32]. Passage 6–8 MSC were used for all experiments described. LEW MSC derived from the bone marrow were positive for MHC class I (RT1A^l^), Thy1 (CD90) and CD73, showed a weak expression of CD80 but lacked the expression of MHC class II, CD4, CD25, CD45, and CD49b as shown by flow cytometry. The resultant MSC had a spindle-shaped fibroblastic morphology and maintained their multilineage differentiation potential by maintaining their ability to differentiate into osterogenic, chondrogenic and adipogenic lineages when cultured in various specific differentiation media.

### Cell preparation

Splenocyte (SC) suspensions were obtained by passing the mouse spleen through a 70-μm cell stainless steel strainer, and erythrocytes were depleted by hypotonic lysis. T cell-depleted SC (act as APC) were enriched from pooled spleens by positive selection via magnetic-activated cell sorting (MACS) using a Pan T Cell Isolation Kit (Miltenyi Biotec). CD4^+^ T cells were enriched from the same pool by negative selection via MACS using a CD4^+^ T Cell Isolation Kit (Miltenyi Biotec). CD4^+^CD25^−^ T cells were sorted by antibody cell sorting on an FACS Vantage SE with a FACS Diva Cell Sorter (BD Bioscience) to >99 % purity.

### Antibodies and FACS analysis

The following monoclonal antibodies (mAbs) used: anti-rat-Nrp-PE, anti-mouse-PD-L1-PE (eBioscience, San Diego, CA), anti-mouse/rat CD28-PE (Santa Cruz, CA), anti-rat CD4-FITC, anti-rat CD25-PE, anti-rat Foxp3-PE-cyanine 5 (eBioscience, San Diego, CA, USA). After membrane and intracellular staining, cells were analyzed on FACS CantoII using the BD FACSDiva v8.0 software.

### Production of lentivirus

Lentivector (FUGW) and packaging (psPAX2) and envelope plasmids (pMD2.G) were obtained from Addgene. The cDNA encoding full-length human Foxp3 (NM_014009.3) was amplified by RT-PCR from the cDNA of adult peripheral cells using specific primers, and digested with restriction enzymes subcloned to lentiviral vector backbone plasmid FUGW. To produce the recombinant lentivirus carrying Foxp3 (Foxp3) and control GFP (FUGW), the recombinant plasmid and vector were co-transfected with packaging and enveloping plasmids into 293FT cells by calcium phosphate transfection. The culture supernatant containing the virus was collected and centrifuged at 15,000 rpm/min for 10 min to remove debris, and then transferred into a 36-mL ultracentrifuge tube for ultracentrifugation at 25,000 rpm/min for 3 h. The pellet containing lentivirus was resuspended. MSC were infected with the appropriate lentivirus where gene transfer efficiency reached at least 80 %.

### Animals and experimental groups

LEW (MHC haplotype: RT1^1^),ACI (MHC haplotype: RT1^a^) and BN (MHC haplotype: RT1^n^) rats weighing approximately 250 g were obtained from the Model Animal Research Center of Central South University, Changsha, China. LEW or BN rats were used as liver transplant donors and ACI rats served as recipients. A rat non-arterialized orthotopic liver transplantation model was performed according to the techniques previously described by Kamada and Calne [33]. Injections of 2 × 10^6^ MSC, FUGW-MSC or Foxp3-MSC into the portal vein were carried out immediately after liver transplantation. ACI recipients were randomly assigned to 7 groups (Table 1): group 1, untreated group; group 2, treated with MSC; group 3, treated with Foxp3-MSC; group 4, treated with FUGW-MSC; group 5, treated with Foxp3-MSC (2 × 10^6^) plus 15.0 mg/kg of rat anti-mouse CD25 mAb on days −2, 0, 2 after liver transplantation, intraperitonially (IP) and group 6 treated with Foxp3-MSC (2 × 10^6^) plus rat control IgG; group 7, treated with 2 × 10^6^ third party BN Foxp3-MSC and group 8, treated with 2 × 10^6^ recipient-derived ACI Foxp3-MSC.

**Table 1:** Experimental groups and liver allograft survival in ACI recipients

Group	n	Injected cells	Graft survival (days)	MST (days)
1	6	No	11, 13 × 2, 14, 15, 16	13.5
2	8	Lewis MSC	16, 18, 20, 21 × 3, 24, 26	21.0*
3	8	Lewis Foxp3-MSC	63, >100 × 7	100*^,†^
4	6	Lewis FUGW-MSC	18 × 2, 20 × 2, 21, 23	20.0*
5	6	Lewis Foxp3-MSC + α-CD25	12, 14 × 3, 16, 18	14.0
6	6	Lewis Foxp3-MSC + IgG	>100 × 6	100*^,†^
7	6	BN Foxp3-MSC	17, 19, 25, 26, 30, 35	25.5*
8	6	ACI Foxp3-MSC	31, 35, 41, 73, >100 × 2	57.0*^,†,††^

* *p* < 0.05 vs untreated group; ^†^ *p* < 0.05 vs MSC group; ^††^ *p* < 0.05 vs Foxp3-MSC group

### Flow cytometry analysis

Cells were analyzed on a flow cytometer (BD Bioscience) according to a previously reported method [34]. For intracellular staining, cells were fixed and permeabilized with Cytofix/Cytoperm (BD PharMingen). The following mAbs were included: anti-rat CD25-PE, anti-rat CD4-FITC, and anti-rat Foxp3-PE-cyanine 5 (eBioscience, San Diego, CA, USA). As to the Foxp3 antibody, an isotype-matched control was used to determine the background. Tregs were identified as CD4^+^CD25^+^Foxp3^+^ triple-positive cells and were expressed as percentages in SC.

### MLR and proliferation studies

MLR and proliferation studies were performed as previously described [7]. CD4^+^ T cells were used as responders (0.5 × 10^6^ cells) in the MLR. We used 4000-rad irradiated SC (0.5 × 10^6^ cells) as stimulators. Cocultures also contained MSC from Lewis rats at ratios of 1:1 and 1:100 in RPMI medium (Gibco Invitrogen, Karlsruhe, Germany) supplemented with 10 % FBS with 800 U/mL rat interleukin-2 (IL-2; BD Bioscience, Bedford, MA, USA). Transwell inserts were used in some wells (0.4-μm pore size; BDFalcon, San Jose, CA, USA). CD4^+^ T cells were cultured in the well beneath the insert. Cell proliferation was determined by pulsing the cells with [3H]thymidine during the last 14–16 h of culture and measuring the radioactivity incorporated by liquid scintillation counting. Proliferative response was expressed as △cpm.

### Antigen-specific tolerance

Long-term ACI liver allograft recipients received full-thickness skin grafts (1 × 1 cm^2^) from LEW donor and BN rat strains on postoperative day (POD) 100 and were monitored daily (n = 3). Rejection was defined as scar formation or epidermis sloughing or both.

### Histology and liver function post-orthotopic liver transplantation

Samples of liver tissue for histopathology were fixed by immersion in 4 % buffered formalin, embedded in paraffin, sectioned, and stained with hematoxylin-eosin. The histological findings of hematoxylin-eosin staining were graded according to the Banff scheme and rejection activity index (RAI) was calculated from the three individual scores [35, 36]. For the evaluation of the engraftment of GFP-MSC in the liver, tissues from GFP-labeled, MSC infused Lewis donor were rapidly frozen, sectioned on a cryostat (6 μm), fixed with acetone, and analyzed directly by fluorescence microscopy. For each tissue, three nonconsecutive sections were analyzed and GFP^+^ cells in each section were counted. Immunohistochemistry (IHC) was performed with anti-rat Foxp3 mAbs (1:100) according to the procedures described in the BioLegend protocol. The levels of alanine aminotransferase (ALT), aspartate aminotransferase (AST) and total bilirubin (TBIL) were measured with an autobiochemical analyzer (CX7; Beckman Coulter, Inc., Brea, CA, USA).

### CD25^+^ cell depletion

CD25^+^ cells Deplation in vivo was performed as previously described [12]. Briefly, mice received 15 mg/kg of rat anti-mouse CD25 mAb (clone PC-61, BioXcell) on days −2, 0, 2 after transplantation, CD25^+^ cell depletion was confirmed on POD7 by staining splenocytes. Rat IgG1 injections at the same time point to reciprocal recipients served as isotype-matched controls.

### Statistics

Allograft survival among experimental groups was compared using log-rank (Mantel-Cox) testing. Statistical analysis for multiplicity was conducted using a one-way ANOVA or the Student *t*-test. Statistical Package for the Social Sciences for Windows (Version 14.0; SPSS Inc.) software was used for all statistical analyses, and *p* < 0.05 was considered statistically significant.

## Results

### Successful gene transfer of foxp3 in MSC and Foxp3-MSC engraftment in liver allograft

**Fig. 1:**
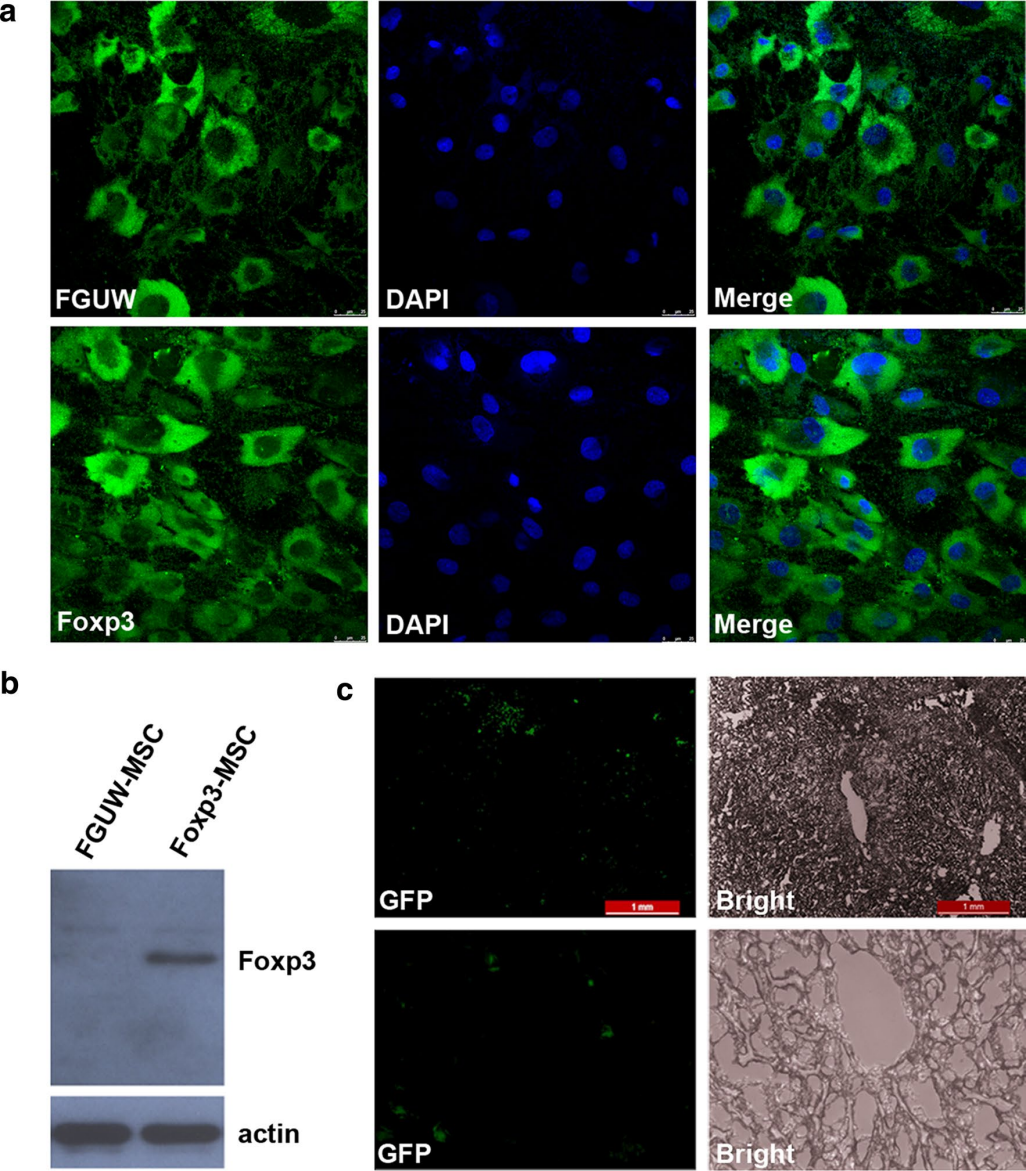
Successful gene transfer of Foxp3 in MSC and Foxp3-MSC engraftment in liver allograft. **a** MSC were infected with control lentivirus (FUGW, *Green*) and GFP-Foxp3 lentivirus (Foxp3, *Green*), Nucleus were stained by DAPI (*Blue*). MSC were efficiently transduced with >95 % GFP-positive cells. **b** The expression of Foxp3 was detected by western blot in Foxp3-MSC and FUGW-MSC lysis at 96 h after infection. The production of Foxp3-GFP fusion protein was confirmed in Foxp3-MSC lysis compared with FUGW-MSC lysis. **c** Liver allograft sections were analyzed by fluorescence microscopy for the presence of GFP-Foxp3-MSC, substantial numbers of GFP-Foxp3-MSC liver graft were observed in liver allograft (*upper panel*, original magnification ×400; *lower panel*, original magnification ×100)

To confirm the successful gene transfer of Foxp3 in MSC through the lentivirus, the transduction efficiency and expression of Foxp3 were examined in Foxp3-engineered MSC cell lysates. MSC were efficiently transduced with >95 % GFP-positive cells (Fig. 1a). Western blot analyses of cell lysis confirmed the production of Foxp3-GFP fusion protein in Foxp3 engineered MSC compared with MSC transfected with an empty vector 96 h after infection (Fig. 1b).

We next evaluated Foxp3-MSC engraftment in liver grafts by fluorescence microscopy. As demonstrated in Fig. 1c, we detected substantial numbers of GFP-Foxp3-MSC (approximately 10–20 cells per section) liver graft of the recipients on POD7 after intraportal injection of allogeneic GFP-Foxp3-MSC. These finding suggests that Foxp3-MSC successful locates in the liver allograft.

### Foxp3 transduction improved the immunosuppressive capacity of MSC and Foxp3-MSC mediated immunomodulation involves cell–cell contact mechanisms

**Fig. 2:**
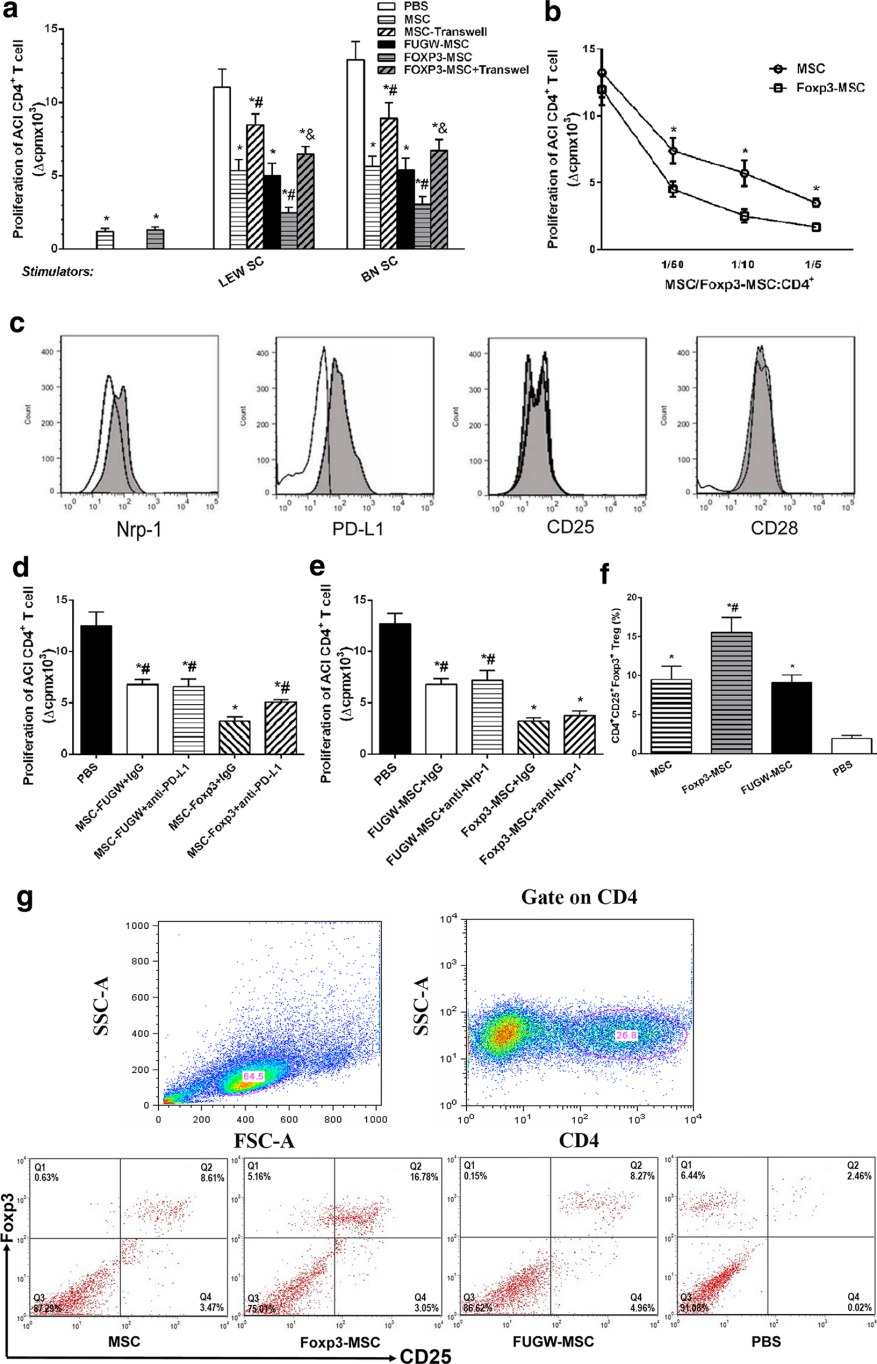
Foxp3 transduction improved the immunosuppressive activity of MSC and induced the expansion of Foxp3^+^ Tregs. **a** The immunogenicity of Foxp3-MSC and effect of Foxp3-MSC on T cell proliferative response in vitro. Results are mean ± SE of three independent experiments. *p < 0.05 vs PBS; ^#^ *p* < 0.05 vs MSC; ^&^ *p* < 0.05 vs Foxp3-MSC. **b** Foxp3 transduction significantly improved the immunosuppressive activity of MSC. Results are mean ± SE of three independent experiments. *p < 0.05 vs MSC. **c** The expression of cell surface molecule in MSCs altered by Foxp3 transduction. Isotype FUGW-MSC are represented by open histograms and Foxp3-MSC by closed histograms. **d**, **e** Blocking experiments with neutralizing mAb of Nrp-1 and PD-L1 on CD4^+^ T cell proliferative response. Results are representative of three independent experiments. *p < 0.05 vs PBS; ^#^ *p* < 0.05 vs Foxp3-MSC + IgG. **f** Frequency of CD4^+^CD25^+^Foxp3^+^ T cells. Results are representative of five independent experiments. *p < 0.05 vs PBS; ^#^ *p* < 0.05 vs MSC. **g** Representative *dot plots* of CD25 and Foxp3 staining of CD4^+^-gated cells. Percentages of CD4^+^CD25^+^Foxp3^+^ T cells are indicated in the *top right corners* of each *dot plot*

To evaluate the immunogenicity of MSC and Foxp3-MSC, as shown in Fig. 2a, MSC and Foxp3-MSC was significantly lower than the proliferation induced by mature splenocytes from LEW rats (Fig. 2a). Actually, ACI CD4^+^ T cell proliferation against LEW MSC or Foxp3-MSC was negligible.

To investigate the effects of Foxp3-engineered MSC on immunosuppressive activity, MLR and proliferation studies were performed. LEW MSC or Foxp3-MSC (MSC/Foxp3-MSC: CD4^+^ T cells = 1:10) significantly lowered the proliferative response of allogeneic ACI CD4^+^ T cells elicited by SC from the same MSC donor strain compared with PBS control group (Fig. 2a). LEW MSC or Foxp3-MSC at the same concentration also significantly reduced the proliferation of allogeneic ACI CD4^+^ T cells to third-party SC from BN rat (Fig. 2a). Furthermore, Foxp3-MSC treatment was significantly lower than the proliferation of allogeneic ACI CD4^+^ T cells induced by SC from the same donor strain or third-party BN rat compared with MSC (Fig. 2a). To further test the effects of Foxp3-engineered MSC on immunosuppressive activity. ACI CD4^+^ T cells were cultured in MLR with allogeneic LEW SC in the presence of graded doses of LEW MSC or Foxp3-MSC. Both MSC and Foxp3-MSC were able to suppress the proliferation of allogeneic CD4^+^ T cells to alloantigens in a dose-dependent manner and Foxp3-MSC showed higher immunosuppressive power when compared with MSC (Fig. 2b). Therefore, both Foxp3-MSC and MSC are not immunogenic and donor specific, Foxp3-MSC share the similar immunological properties with MSC and Foxp3 transduction significantly improved the immunosuppressive capacity of MSC.

MSC-mediated immunomodulation involves both cell contact–dependent and cell contact–independent mechanisms mediated through the release of soluble factors. To determine the role of cell contact, trans-well experiments were performed, in which direct contact between T cells and allogeneic Foxp3-MSC was prevented, Fig. 2a shows that when cell–cell contact is prevented, similar to MSC, the suppression effect of Foxp3-MSC on the proliferation of ACI CD4^+^ T cells is significantly alleviated (*p* < 0.05 vs Foxp3-MSC). Therefore, the cell–cell contact is required to immunosuppresion activity of Foxp3-MSC.

We further investigate the phenotype of MSC altered by Foxp3 transduction. Foxp3-MSC and FUGW-MSC were isolated using a FACS and CD25, CD28, Nrp-1, and PD-L1 regulated by transcription factors Foxp3 [30, 37] were detected by FCM. MSC overexpressing Foxp3 increased the expression of cell surface molecule Nrp-1 and costimulatory molecule PD-L1 compared with FUGW-MSC (Fig. 2c); however, no difference between the two groups in the expression of cell surface molecule CD25 and costimulatory molecule CD28 was found (Fig. 2c). To study the role of the Nrp-1 and PD-L1 in mediating suppression of CD4^+^ T cell proliferation by LEW Foxp3-MSC, blocking experiments were performed using 10 μg/ml of neutralizing antibodies against PD-L1 or Nrp-1. Foxp3-MSC treated with anti-PD-L1 significantly increased the proliferation of allogeneic ACI CD4^+^ T cells elicited by SCs from LEW compared with Foxp3-MSC with IgG, while the presence of 10 μg/ml of anti-PD-L1 antibody did not produce significant effect on the FUGW-MSC mediated inhibition of cell proliferation (Fig. 2d). Thus, the increased expression of PD-L1 in Foxp3-engineered MSC may be associated with its immunosuppressive capacity. On the other hand, the blockade of Nrp-1 did not produce significant effect on the FUGW-MSC or Foxp3-MSC mediated inhibition of CD4^+^ T cells proliferation (Fig. 2e).

### Foxp3 transduction induced the expansion of Foxp3^+^ Tregs in vitro

To determine whether or not the immunoregulatory actions of Foxp3-MSC were associated with the expansion of Tregs, CD4^+^ T cells were cocultured with MSC, Foxp3-MSC, or FUGW-MSC for 7d in the presence of APC and anti-CD3 mAb, and their FCM results were analyzed. Figure 2f, g shows that the MSC markedly increased the percentage of CD4^+^CD25^+^Foxp3^+^ T-cell population after 7 days of cocultures with CD4^+^ T cells, Furthermore, the percentage of CD4^+^CD25^+^Foxp3^+^ Tregs in the Foxp3-MSC group was significant higher than that in the MSC group.

### Foxp3-MSC monotherapy achieves donor-specific liver allograft tolerance

**Fig. 3:**
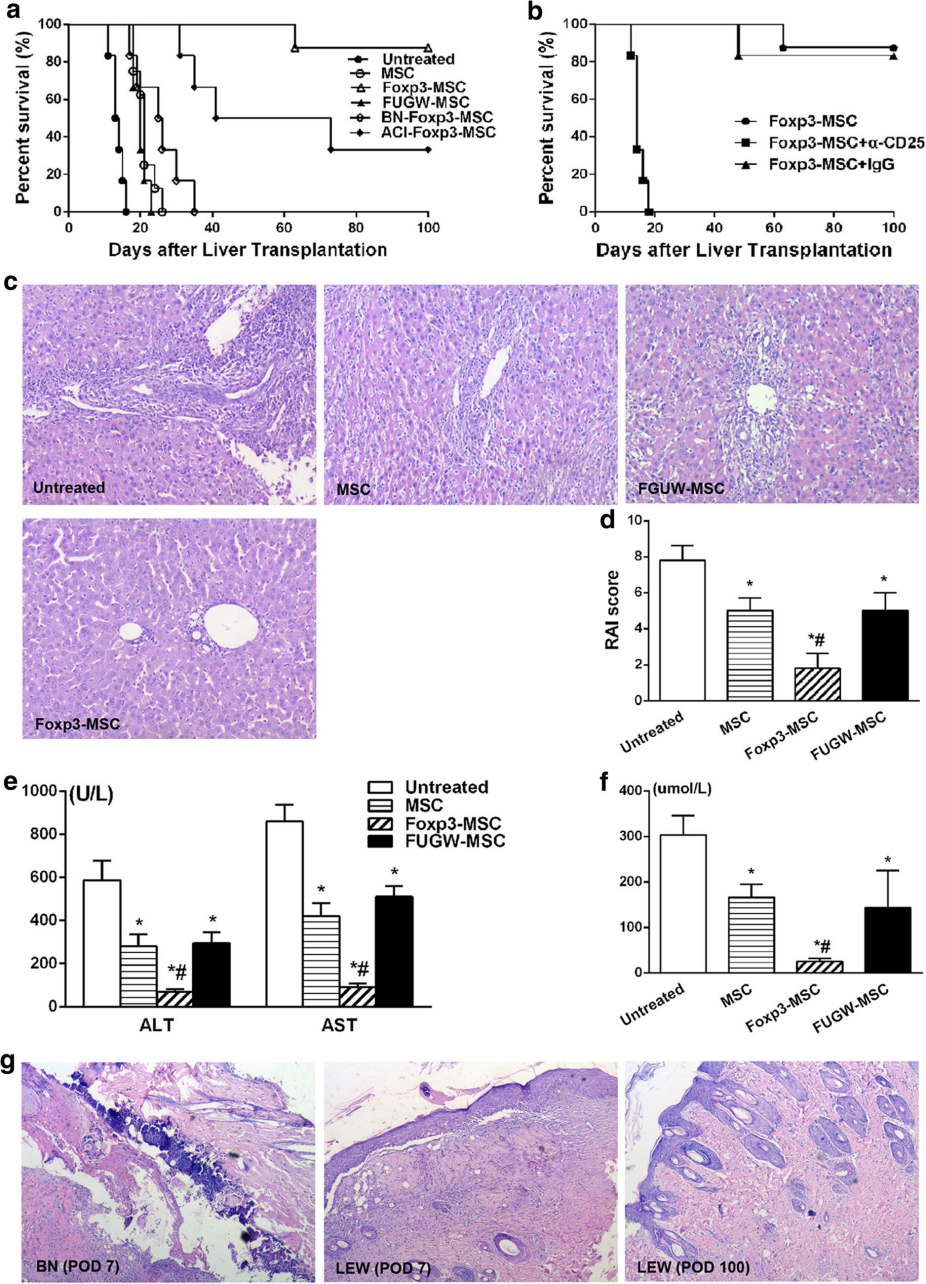
Foxp3-MSC monotherapy achieves donor-specific liver allograft tolerance. **a** Kaplan–Meier survival curves for untreated, donor-derived MSC, FUGW-MSC and Foxp3-MSC, recipient-derived Foxp3-MSC and third party Foxp3-MSC groups. **b** Kaplan–Meier survival curves for the Foxp3-MSC, Foxp3-MSC+IgG and Foxp3-MSC+α-CD25 groups. **c** Histology of liver allografts in ACI recipients on POD 7 (H&E, original magnification ×100). **d** RAI scores according to the Banff scheme. Results are mean ± SE of five independent experiments. *p < 0.05 vs untreated; ^#^ *p* < 0.05 vs MSC. **e**, **f** Changes in the serum ALT, AST and TBIL concentrations in all 4 groups 7 days after LT. Results are mean ± SE of three independent experiments. *p < 0.05 vs untreated; ^#^ *p* < 0.05 vs MSC. **g** Representative skin biopsy samples were obtained on day 7 and 100 after skin transplantation (H&E, original magnification ×100)

To elucidate the tolerogenic effect of Foxp3-MSC on liver transplantation, MSC、Foxp3-MSC and FUGW-MSC from donor Lewis rat were injected into ACI recipients immediately after receiving a orthotopic Lewis liver graft. Untreated allografts were rejected with a median survival time (MST) of 13.5 days (Table 1; Fig. 3a) and MSC or FUGW-MSC treated recipients significantly prolong graft survival with a MST of 21.0 and 20.0 days respectively (Table 1; Fig. 3a). Importantly, donor-derived Foxp3-MSC treated recipients achieved indefinite graft survival (>100 days) with almost normal histology (Fig. 3a, c). In contrast, third party Foxp3-MSC did not influence graft survival as compared to animals receiving donor-derived MSC (Table 1, group 7, *p* = 0.16, compared to group 2), whereas two out of six animals treated with recipient-derived Foxp3-MSC accepted their grafts long-term (Table 1, group 8, *p*<0.05, compared to group 2 and group 3). Hence, donor-derived Foxp3-MSC is most effective for tolerance induction.

Next, we examined the pathology and function of liver allografts. As shown in Fig. 3c, severe acute rejection was present in the untreated groups at day 7 postoperatively; moderate acute rejection with milder vacuolation of hepatic cells and less inflammatory cell infiltration were observed in rat treated with MSC or FUGW-MSC compared to untreated group. However, Foxp3-MSC treatment achieved almost normal histology on POD 7. At the same time, RAI score of Foxp3-MSC group was markly lower than that in untreated, MSC or FUGW-MSC control group (Fig. 3d). The serum concentrations of liver enzymes (ALT and AST) and TBIL were significantly decreased after Foxp3-MSC treatment compared with those after treatment with untreated, MSC or negative control lentivirus (Fig. 3e, f).

To study whether the long-term graft acceptance was associated with donor-specific tolerance, Full-thickness skin transplantation was also performed, skin grafts from either LEW donor or BN third-party rat were transplanted to long-term surviving ACI recipients on POD 100, BN third-party skin grafts were rejected within 14 days, while LEW donor skin grafts were survived over 100 days (data not shown). A histological examination of skin grafts were completed on POD 7 after skin transplantation, intense infiltration of inflammatory cells was observed in the epidermis and dermis of the BN third-party skin grafts (Fig. 3g) but not in LEW donor skin grafts, indicating that donor-specific tolerance was achieved in LEW Foxp3-MSC treated liver allograft recipients.

### Foxp3-MSC increase CD4^+^CD25^+^Foxp3^+^ Tregs frequencies and generate a state of CD4^+^CD25^+^Foxp3^+^ Tregs-dependent tolerance

**Fig. 4:**
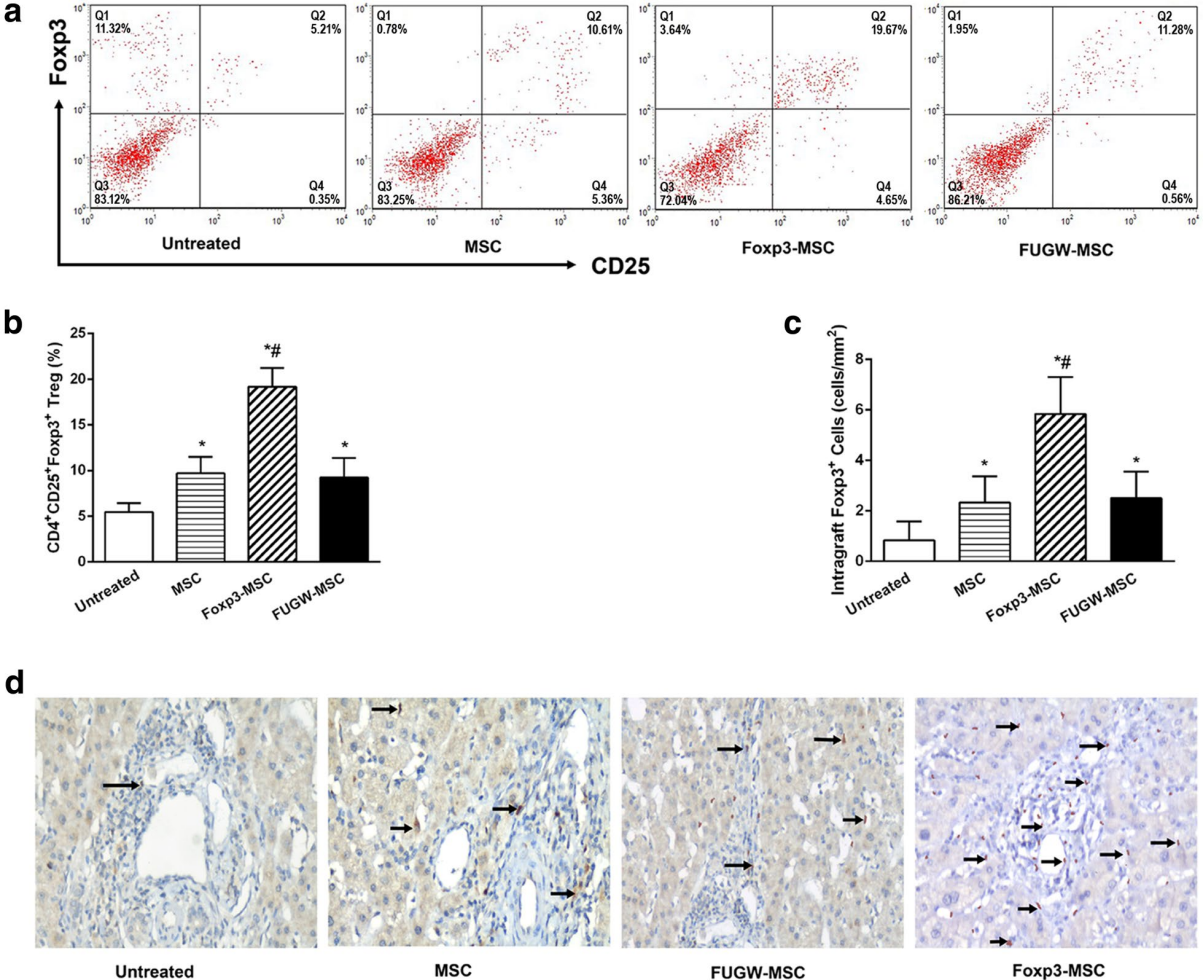
Frequency of Tregs generated in ACI recipients. **a** Representative *dot plots* of splenic Tregs. Percentages of CD4^+^CD25^+^Foxp3^+^ T cells are indicated in the *top right corners* of each *dot plot*. **b** Graphic results of frequency of splenic CD4^+^CD25^+^Foxp3^+^ T cells. Results are representative of five independent experiments. *p < 0.05 vs untreated; ^#^ *p* < 0.05 vs MSC. **c** Graphical representation of numbers of Foxp3^+^ T cells (cells/mm^2^) within the liver allografts. The results are representative of six separate experiments. *p < 0.05 vs untreated; ^#^ *p* < 0.05 vs MSC. **d** Representative immunohistochemistry staining of intragraft Foxp3^+^ T cells. (IHC, original magnification ×100)

Because CD4^+^CD25^+^Foxp3^+^ Tregs proportions had previously been confirmed to increase on co-culturing naïve T cells with Foxp3-MSC in vitro (Fig. 2f, g), we examined whether CD^+^CD25^+^Foxp3^+^ T cells contributed to liver allograft acceptance in Foxp3-MSC treated allograft recipients. Our data showed that tolerant recipients had a significant increase in splenic CD4^+^CD25^+^Foxp3^+^ Tregs frequency compared with other control groups (Fig. 4a, b). For confirmation, immunohistochemical analysis of Foxp3^+^ cells was also performed in liver allograft tissues to document Tregs recruitment at the graft site. Significant numbers of intragraft positive staining Foxp3 cells were found in Foxp3-MSC treated recipients compared with untreated, MSC or FUGW-MSC treated recipients on POD 7 (Fig. 4c, d). These results indicate that graft tolerance induced by Foxp3-MSC is associated with the accumulation of Foxp3^+^ Tregs in the graft.

To understand the immunological role of CD4^+^CD25^+^Foxp3^+^ Tregs behind Foxp3-MSC mediated long-term graft acceptance, CD25^+^ T cells were depleted by in vivo administration of an α-CD25 monoclonal antibody (mAb) on days −2, 0, 2 after transplantation, and CD4^+^CD25^+^Foxp3^+^ T cells frequencies were confirmed less than 0.9 % of the mononuclear cell population on POD7. As shown in Table 1 and Fig. 3b, although having received Foxp3-MSC treatment, liver grafts were rejected in 20 days after CD25^+^ T cells depletion. In contrast, Foxp3-MSC recipients received isotype control IgG maintained long-term liver graft survival. These data further indicating a critical role for Foxp3^+^ Tregs in Foxp3-MSC inducing tolerance.

## Discussion

MSC have recently emerged as promising candidates for cell-based immunosuppression/tolerance and can be easily obtained and propagated in culture [7–9]. However, treatment with MSC alone only prolonged allograft survival but could not induce allograft tolerance in rodent transplantation models [8, 9, 11, 13, 38, 39]. MSC also only be applied as a complement to standard immunosuppressive therapy in a clinical setting [14, 15]. Previous studies have been performed to show that gene modification of MSC, including the incorporation of exogenous genes such as IL-10 [40], hepatocyte growth factor [41], IL-7 [42] successfully improve MSC therapeutic ability. In the present study, we intended to enhance the tolerogenic effect of MSC by overexpressing gene Foxp3. Our results show that Foxp3-MSC is not donor specific/immunogenic, Foxp3 transduction significantly improve the immunosuppressive capacity of MSC, Foxp3-MSC suppressive effect on the proliferation of CD4^+^ T cells is contact dependent and associated with PD-L1 upregulation. More importantly, Foxp3-MSC monotherapy achieves donor-specific liver allograft tolerance, the tolerogenic potential of Foxp3-MSC is associated with the expansion of CD4^+^CD25^+^Foxp3^+^ Tregs.

Foxp3 is essential for specifying the Foxp3^+^ Treg cell lineage during development, and continued expression of Foxp3 in mature Treg cells is necessary for suppressive function [43]. Deletion of Foxp3 in fully differentiated mature Treg cells results in the deregulation of its target genes and the loss of suppression function [44]. Previous studies have identified Foxp3 target genes and reported a large number of Foxp3-bound genes that are up- or down-regulated in Foxp3^+^ T cells, suggesting that Foxp3 acts as both a transcriptional activator and a repressor. Furthermore, Foxp3 probably sets up a transcription factor network controlling the overall functional program of Tregs [29, 30]. The present study utilized a lentivirus vector to modify MSC to overexpress the therapeutic gene Foxp3, the findings suggested that the immunosuppressive effect of rat Foxp3-MSC was dose dependent and not donor specific/immunogenic in vitro. Indeed, Foxp3-MSC/MSC inhibited the proliferative response of both autologous and allogeneic CD4^+^ T cells to either donor or third-party alloantigens in MLR, which is consistent with most published studies [7–9, 11–13]. Moreover, Foxp3 transduction significantly improved the immunosuppressive capacity of MSC, Further analyses indicated that Foxp3-MSC mediated immunomodulation involved cell–cell contact mechanisms. Using neutralizing antibodies specific for PD-L1, we showed that the mechanisms by which Foxp3-MSC mediated enhanced immunosuppressive capacity was associated with costimulatory molecule PD-L1 of MSC altered by Foxp3 transduction.

Programmed death-1 (PD-1) is a molecule expressed on various cell types, including a subset of thymocytes and activated T and B cells that plays an important role in the negative regulation of immune responses and the maintenance of peripheral tolerance [45]. PD-1 ligand, PD-L1, broadly expressed in different tissues, has been shown to be further induced by exposure to interferon (IFN)-γ on several tumour cell lines and MSC [45, 46]. The exact immune functions of PD-L1 are not fully understood. With respect to immunosuppression mediated by MSC, our findings are consistent with previous study that murine MSC inhibit lymphocyte proliferation by activation of the PD-L1-mediated signaling pathway [47]. The study showed that MSC inhibited the activation and proliferation of murine lymphocytes via engagement of the inhibitory molecule PD-L1 to its cognate receptor on target immune cells, and MSC-mediated inhibition could be blocked by anti-PD-L1 antibody [47, 48]. Recently, it has been shown that IFN-γ acts directly on MSC leading to an up-regulation of PD-L1, and PD-L1 knockdown abolished MSC suppressive properties [49]. In vivo, the combination therapy of MSC with PD-L1 expression and rapamycin induced immune tolerance to cardiac allografts, while the blockade of PD-L1 on MSC with monoclonal antibody abrogated the combination therapy-induced immune tolerance [50]. The PD-1/PD-L1 pathway is essential for costimulatory blockade-induced allograft tolerance, which reflects the roles of the pathway in controlling T effector cell proliferation, as well as the induction of T-cell anergy [51]. However, whereas English and collaborators reported that PD-1 pathway was not essential for MSC mediated immunosuppression [52]. The reasons for these differences are most likely due to the use of different stem cell populations and immune cells. Indeed, the authors used not only MSC with different phenotypes but also heterogeneous population of immune cells such as splenocytes or lymphocytes. Taken together our results demonstrated the cell-to-cell contact depend mechanism of Foxp3-MSC immunosuppression on CD4^+^ T cells is most likely to involve the up-regulation of PD-L1.

In order to determine how Foxp3-MSC modulated Tregs in our study, we cocultured Foxp3-MSC/MSC with CD4^+^ T cells in vitro (MLR) to mimic some of the conditions in vivo. Co-culture of CD4^+^ T cells with Foxp3-MSC results in a shift towards a Tregs phenotype compared with MSC group. MSC are known to express cell adhesion molecules [53] and several authors have confirmed that direct interaction with target cells is a requirement for their overall immunosuppressive effect [53, 54]. However, there is currently a notable lack of information on the importance of, and mechanisms underlying, the induction of Tregs by MSC via contact-dependent mechanisms [55]. Studies have also shown other factors may be able to substitute for cell-to-cell contact in some situations, as cell-to-cell contact was essential when MSC were co-cultured with purified CD4^+^ T cells, but this direct interaction was not essential when MSC were exposed to an un-fractionated mononuclear cell population [56]. These findings are consistent with a cell contact-dependent mechanism as suggested by the phenotype of MSC altered by Foxp3 transduction experiments in which Foxp3 transduction led to an up-regulation of PD-L1 on MSC. In the present study, the importance of Tregs in induction of transplant tolerance was also supported by high frequencies of Foxp3^+^ Tregs in the spleens and liver allografts of tolerant recipients. Antibody-mediated depletion of CD25^+^ cells abrogated liver allograft tolerance despite administration of Foxp3-MSC, further indicating a critical role for Tregs. These results suggested that the induction and maintenance phases of tolerance after donor-derived Foxp3-MSC infusion in rats are associated with the emergence of functional Tregs.

In this study, Foxp3-MSC/MSC were well detected in the recipient liver allografts after portal vein injection, suggests that Foxp3-MSC/MSC migration and/or proliferation is influenced by tissue injury and/or inflammatory signals. These results are also in line with previous studies indicating that the liver is an immune privileged organ for tolerance induction by donor cell infusion [7, 9, 15]. Foxp3-MSC treatment significantly lowered the proliferation of allogeneic ACI CD4^+^ T cells to SC from the same donor strain or third-party BN rat. However, in our model third party MSC did not induce graft tolerance, whereas two out of six animals treated with recipient-derived Foxp3-MSC accepted their grafts long-term (Table 1; Fig. 3b). These findings are consistent with previous studies [7, 32] and implicate that MSC cannot be used from arbitrary donors in a clinical transplantation setting. Understanding the mechanism behind MSC mediated tolerance most likely helps to augment the efficiency of recipient-derived MSC, which makes this strategy clinically applicable to cadaveric organ transplantation.

## Conclusions

In this study, Foxp3 transduction significantly improved the immunosuppressive activity of MSC, although immunomodulatory mechanisms of Foxp3-MSC remain unclear, Foxp3-MSC suppressive effect on the proliferation of CD4^+^ T cells is contact dependent and associated with PD-L1 upregulation. Foxp3 transduction induced the expansion of Foxp3^+^ Tregs and they played an important role in Foxp3-MSC induced liver allograft tolerance. Foxp3-engineered MSC therapy seems to be a promising and attractive cell therapy approach for inducing immunosuppression or transplant tolerance.

## Abbreviations

ALT: alanine aminotransferase
APC: antigen-presenting cell
AST: aspartate aminotransferase
CFSE: carboxyfluorescein diacetate succinimidyl ester
DMEM: Dulbecco’s modified Eagle’s medium
FBS: fetal bovine serum
Foxp3: transcription factor forkhead box P3
Foxp3-MSC: Foxp3 modified MSC
FUGW-MSC: FUGW modified MSC
mAb: monoclonal antibody
IHC: immunohistochemistry
MACS: magnetic-activated cell sorting
MLR: mixed lymphocyte reaction
MSC: mesenchymal stem cells
MST: mean survival time
Nrp-1: neuropilin-1
OD: optical density
PBS: phosphate buffer solution
PD-1: programmed cell death-1
PD-L1: programmed death ligand 1
PE: phycoerythrin
POD: postoperative day
RAI: rejection activity index
SC: splenocytes
TBIL: total bilirubin
Tregs: regulatory T cells
